# State Emotional Clarity Is an Indicator of Fluid Emotional Intelligence Ability

**DOI:** 10.3390/jintelligence11100196

**Published:** 2023-10-10

**Authors:** Nathaniel S. Eckland, Renee J. Thompson

**Affiliations:** Department of Psychological and Brain Sciences, Washington University in St. Louis, St. Louis, MO 63130, USA

**Keywords:** emotional clarity, emotional intelligence, experience sampling

## Abstract

Emotional clarity is one facet of emotional intelligence that refers to one’s meta-understanding of and ability to identify and describe feelings. The existing research has largely focused on trait emotional clarity and its benefits for greater psychological well-being, more successful emotion regulation/coping, and diminished psychopathology. Researchers have begun to examine state or momentary emotional clarity in daily life. In this paper, we situate emotional clarity within the larger literature on emotional intelligence abilities. Then, we argue that state clarity relies on the ability to incorporate information from the dynamic contexts that emotions unfold in and should more closely reflect one’s emotional intelligence ability relative to traditional trait measures. In addition, we review and make recommendations for measuring state emotional clarity in daily life and propose future research directions, focusing on how state emotional clarity could inform the study of emotion regulation, decision making, and goal pursuit in daily life.

## 1. Introduction

Conceptions of emotional intelligence frequently involve sets of abilities related to one’s own and others’ emotions. Among the abilities proposed to comprise emotional intelligence, abilities related to perceiving emotions, understanding emotions, and using/regulating emotions are some of the most frequently cited (Salovey et al. 1995; Mayer et al. 2002; Joseph and Newman 2010). Emotional clarity is the ability to identify and describe one’s emotional experiences (Gohm and Clore 2000; Salovey et al. 1995) and is thought to encompass one’s meta-perceptions about their emotions (Boden and Thompson 2017). We argue that emotional clarity should be considered an integral ability for emotional intelligence and that measures of state emotional clarity will give the greatest insights into one’s access to that ability in daily life. 

Like emotional intelligence, emotional clarity has received attention across disciplines in psychology, including, but not limited to, clinical, cognitive, personality, social, and industrial/organizational psychology. Though emotional clarity can be understood as a standalone construct, it is also a dimension of several multidimensional constructs, including alexithymia (i.e., a condition characterized by difficulty identifying and describing feelings; Bagby et al. 1994), emotional awareness (Boden and Thompson 2015; Eckland and Berenbaum 2021), and sometimes emotional intelligence (or “perceived emotional intelligence”; Salovey et al. 2002). The broad interest in emotional clarity is unsurprising given its importance for processing emotional experiences (Gohm 2003) and links to several healthy outcomes (e.g., subjective well-being; Gohm and Clore 2002). 

In the present paper, we first discuss why emotional clarity should be considered a key ability involved in emotional intelligence. Second, we propose that the emerging research on state emotional clarity suggests it is an indicator of emotional intelligence ability. Third, we review and make recommendations for measuring state emotional clarity. Fourth, we posit that intra-individual variation in state emotional clarity reflects access to emotional intelligence ability and we describe sources of this variation. Finally, we present directions for future research involving state emotional clarity, focusing on emotion regulation, decision making, and goal pursuit. 

## 2. Emotional Clarity and Emotional Intelligence Ability

Several frameworks of emotional intelligence ability, including Mayer et al.’s (2002) four-branch ability model and Joseph and Newman’s (2010) cascading model, cite emotion perception and understanding as key emotional intelligence abilities. Emotion perception has been defined as the ability to recognize emotions in the self, others, and in other stimuli such as art and media (Mayer et al. 2002). Emotion understanding has been defined as the ability to understand and appreciate emotional information, such as how more complex emotions may be blends of more simple emotions and how emotions vary in intensity (Mayer et al. 2002). As a construct, emotional clarity is relevant to both perceiving and understanding emotions. Emotional clarity is thought to involve creating a clear mental representation of one’s emotional experience based on perceived stimuli from the body and external context, which can then be translated from a mental representation into a verbal representation (Hoemann et al. 2021). To be emotionally clear is to have perceived and understood one’s emotions. 

Despite conceptual overlap, measures of emotional clarity (most frequently measured with the Trait Meta-Mood Scale [TMMS]; Salovey et al. 1995) and ability-based emotional intelligence (often assessed by the Mayer-Salovey-Caruso Emotional Intelligence Test [MSCEIT]; Mayer et al. 2002) have tenuous links. Studies find that emotional clarity is either uncorrelated (Lopes et al. 2003) or positively correlated only to a small degree (Koven and Max 2014) with total MSCEIT scores, MSCEIT perceiving, and MSCEIT understanding scores. Emotional clarity, as it is typically measured through self-report, has sometimes been labeled as “perceived” emotional intelligence (Salovey et al. 2002). Perceived emotional intelligence has also been critiqued as being difficult to separate from personality, though some work demonstrates that personality variables account for large amounts of the variance in performance in emotional intelligence ability tests (Fiori and Antonakis 2011; Schulte et al. 2004). There are several possible explanations for this lack of coherence among self-report and performance-based measures. 

In the MSCEIT, emotion perception is tested with two tests: identifying emotions in pictures of faces (the Faces test) and identifying emotions conveyed through pictures of artwork and landscapes (the Pictures test). The understanding facet of emotional intelligence is also measured with two tests: identifying emotions that are intensifications of other emotions (the Changes test) and identifying emotions that are blends of other emotions (the Blends test). Though the emotion perception facet is defined as being the ability to recognize emotions in the self, others, and other stimuli, the MSCEIT only measures the ability to recognize emotions on static faces and in stimuli such as landscapes. Likewise, the emotion understanding tests do not test the ability to identify changes in intensity or blends of emotions in the self. However, this issue is not unique to the MSCEIT. Other tests of emotion perception (e.g., the Geneva Emotion Recognition Test; Schlegel et al. 2014) and emotion understanding (e.g., the Situational Test of Emotion Understanding; MacCann and Roberts 2008) similarly focus on recognizing the emotions of others or identifying how one “should” feel given a hypothetical situation, rather than testing one’s ability to perceive and understand their own emotions. Accurate emotion recognition and clearly perceiving one’s own emotions are modestly linked, but are by no means the same skill (Eckland et al. 2018). Thus, within the current literature on emotional intelligence abilities, a gap exists between what one may know about identifying emotions (i.e., what is measured) versus the experience of identifying emotions in oneself (i.e., what is not measured). 

These emotional intelligence ability tests likely reflect one’s declarative knowledge about emotion categories, but not necessarily one’s procedural knowledge of identifying their own emotions. This is further underscored by work showing that MSCEIT scores are more strongly related to crystallized, versus fluid, intelligence (Farrelly and Austin 2007), indicating that the MSCEIT may be drawing upon acquired knowledge rather than pure ability. Fiori et al. (2014) also found that the MSCEIT tests better discriminate persons with low emotional intelligence, but are likely not challenging enough to persons that are high in emotional intelligence. As evidenced by clinical interventions to increase emotional clarity (Linehan 2015), a conceptual understanding of emotions can be used as a building block for the more challenging skill of perceiving and understanding one’s own emotions as they unfold in day-to-day life. The MSCEIT and other tests of emotional intelligence ability are measures of maximal emotional intelligence performance (i.e., it is a performance measure given under “ideal” conditions) rather than typical performance, which relates to one’s ability/access to abilities in everyday life. Thus, the current emotional intelligence ability measures are likely testing crystalized emotion knowledge, but not how well a person can access, use, and apply that knowledge in the real world. This is also illustrated by Montgomery et al.’s (2010) study of autistic young adults, who did not score significantly different from neurotypical young adults on total MSCEIT emotional intelligence ability, but self-reported significantly lower emotional intelligence. In contrast, measures of emotional clarity focus on one’s perception of emotions in the self under typical conditions (i.e., trait measures) or under current contextual demands (i.e., state measures and states in daily life). 

## 3. Emotional Clarity as an Indicator of Fluid Emotional Intelligence Ability

Fiori and Vesely-Maillefer (2018), Fiori et al. (2021), drawing on the Cattell-Horn-Carol model of crystallized and fluid intelligence (Schneider and McGrew 2012), proposes a distinction between crystallized emotional intelligence ability and fluid emotional intelligence ability. Crystallized emotional intelligence ability is what is captured in tests, such as the MSCEIT, that draw upon declarative knowledge about emotions, whereas fluid emotional intelligence ability involves the processing of emotional information. Ortony et al. (2007) proposed that a fluid component of emotional intelligence is necessary and should include experiential measures rather than measures that more exclusively reflect declarative knowledge about emotions. 

We believe that emotional clarity, reflecting one’s ability to a create a clear mental (and verbal) representation of their emotional experiences, should be considered an indicator of fluid emotional intelligence ability. Empirical evidence suggests that emotional clarity facilitates the healthy processing of emotional experience. Higher emotional clarity has been linked to faster processing of negative emotional information (Fisher et al. 2010). Lower emotional clarity has been linked to indicators of poorer emotional information processing such as less prosocial moral decision making (Koven 2011), reduced meaning in life in the face of existential threat (Abeyta et al. 2015), and difficulty using affective information to inform judgment (Gohm 2003). 

Research has also unambiguously linked trait emotional clarity to a host of psychological processes and outcomes that one would expect a facet of emotional intelligence to be linked to. Low trait emotional clarity has been linked to several indicators of psychopathology, including depression (Boden and Thompson 2015; Eckland et al. 2021; Vine and Aldao 2014), worry (Eckland and Berenbaum 2021; McLaughlin et al. 2007), panic (Park and Naragon-Gainey 2019; Salters-Pedneault et al. 2006; Tull and Roemer 2007), and problematic alcohol use (Vine and Aldao 2014). Higher emotional clarity has been linked to several indicators of well-being, including problem-solving (Gohm and Clore 2002), life satisfaction (Eckland and Berenbaum 2023; Lischetzke et al. 2012), meaning in life (Abeyta et al. 2015), successful down regulation of negative affect (Wilkowski and Robinson 2008), and use of putatively adaptive emotion regulation strategies (e.g., reappraisal and acceptance; Boden and Thompson 2015). 

Despite primarily being studied as a trait, emotional clarity is a dynamic process (Eckland and Berenbaum 2021; Lischetzke et al. 2011; Park and Naragon-Gainey 2019; Thompson and Boden 2019). That is, emotional clarity can fluctuate in daily life, varying over time and across situations for an individual. Trait emotional clarity refers to the extent to which one typically understands their emotions, whereas state emotional clarity refers to the extent to which one clearly understands their emotions at shorter time scales (e.g., emotional clarity over the course of a day, hour, or in the moment). Though abilities are thought to be largely static, and are thus measured through maximal performance, in daily life, persons interact with psychological and environmental contexts that may limit or facilitate their access to these abilities (van Vianen 2018). Zeidner et al. (2008) argued that this also describes emotional intelligence. Though the correlates of trait emotional clarity converge with emotional intelligence ability, the traits measured through self-report involve retrospecting over large swaths of time and can be influenced by other sources, such as one’s self-perceptions (Paulhus and Vazire 2007). In contrast, leveraging methods, such as experience sampling, has allowed researchers to begin to understand how emotional clarity states fluctuate across time and situations, giving insights into how emotions are perceived and understood in daily life. 

We argue that fluctuations in emotional clarity may be especially important to study because they could also reflect differential access to one’s fluid emotional intelligence abilities across various contexts. Reports of state or momentary emotional clarity rely on the abilities to incorporate information from various sources at a given time (e.g., the dynamic contexts that emotions unfold in) and indicate one’s online ability to clearly represent their emotional experiences. 

## 4. Measuring State Emotional Clarity 

To date, only a handful of studies have assessed state emotional clarity in daily life. Below, we review how state emotional clarity has been operationally defined and measured across these studies. We also provide recommendations for measurement and situate these measurement issues within the emotional intelligence ability field.

### 4.1. Direct Measures

Direct measures of examining state emotional clarity involve relatively straightforward self-reporting about one’s experience. These measures prioritize face-validity (i.e., it is clear to the respondent what they are asked to report on; Paulhus and Vazire 2007). In the context of measuring state emotional clarity in experience sampling research, item selection is an important design decision as it is rare to adapt an entire subscale to an experience sampling protocol. Below, we review the current body of available research directly measuring emotional clarity in daily life. 

Eight studies (Bailen et al. 2019; Eckland and Berenbaum 2021; Eckland and English 2023; Eisele et al. 2023; Park and Naragon-Gainey 2019; Springstein et al. 2023; Thompson and Boden 2019; Tuck et al. 2023) have used face-valid items to assess state (e.g., momentary, daily) levels of emotional clarity. In most cases, these items were modified versions of trait items selected for having the highest factor loading on a trait measure of emotional clarity. For example, Thompson and Boden (2019) and Bailen et al. (2019), who utilized the same sample (N = 79), assessed momentary emotional clarity using the item “At the time of the beep, I was clear about my feelings”. They modified the item of the emotional clarity of feelings subscale of the TMMS that had the highest factor loading (Salovey et al. 1995) by adding “at the time of the beep”, and changing the sentence structure to past tense. Springstein et al. (2023), Eckland and English (2023), and Tuck et al. (2023) administered the same item to assess momentary emotional clarity in three experience sampling studies (Springstein et al. 2023: 10 days, N = 277; Eckland and English 2023: 9 days, N = 219; Tuck et al. 2023: 14 days, N = 206). Park and Naragon-Gainey (2019) measured state emotional clarity using event-contingent experience sampling (i.e., participants, N = 129, completed a survey when they had a strong or significant emotion episode) by having participants rate the “extent to which they were able to clearly identify the emotions” during a strong emotion episode. Eckland and Berenbaum (2021) measured emotional clarity during a seven-day daily diary study (N = 212) using the items: “Today my emotions were clear” and “Today I was confused about how I felt”. Finally, Eisele et al. (2023) measured momentary emotional clarity in a two-week experience sampling study (N = 163) with an item that they developed: “I found it difficult to indicate in a number how I was feeling”. As state emotional clarity could refer to many time frames (e.g., momentary/“at the time of the beep”, past hour/“over the last hour”, daily/“today”, during a specific emotion episode, or since the last survey), it is important to consider the time frame that the item stem refers to when designing an experience sampling study. 

To test the assumption that momentary and trait emotional clarity assess the same latent construct, some of the studies described above have reported associations between the trait measures of emotional clarity and state/momentary measures. Thompson and Boden (2019) and Bailen et al. (2019) found a positive, but not statistically significant, relationship between emotional clarity at the momentary and trait level. More specifically, their momentary emotional clarity item was not significantly associated with the trait measure of emotional clarity, which was assessed as recommended by Palmieri et al. (2009). However, Park and Naragon-Gainey (2019) and Eckland and Berenbaum (2021) found moderate to strong associations between trait and state measures. Park and Naragon-Gainey (2019) reported significant associations between trait emotional clarity (using the Toronto Alexithymia Scale [TAS-20]; Bagby et al. 1994) and state emotional clarity during strong emotion episodes. Eckland and Berenbaum (2021) also found a significant association between trait (using the TMMS) and daily emotional clarity. These findings suggest that state and trait measures of emotional clarity are likely assessing the same construct, but there may be some circumstances that produce greater correspondence.

Across these studies, the time-anchors for the state emotional clarity item differed (Thompson and Boden: “at the time of the beep”; Park and Naragon-Gainey (2019): in response to strong emotional event; Eckland and Berenbaum (2021): reflecting over course of day). Emotional clarity should vary in daily life according to when significant or emotional events occur (e.g., Thompson and Boden (2019) found momentary emotional clarity was higher after a significant positive event). Thus, it is possible that heterogeneity in the magnitude of the association between state and trait measures of emotional clarity may be due to the window of time that one is retrospecting over and whether significant or emotional events can be used to ground those ratings. With regard to reporting on emotional experience, Robinson and Clore (2002) found that when reflecting over shorter spans of time, participants rely more on episodic memory, but for longer spans of time participants rely more on semantic memory. It may be that the window of time that one is reporting emotional clarity over could also activate different types of memory (e.g., reporting over a few hours to a day may activate more semantic than recent episodic memory), which could affect the coherence with trait measures (which may rely more on semantic memory). Thus, ratings of emotional clarity in the moment may have less correspondence with trait levels than ratings made by reflecting over longer windows of time. More empirical work is needed to identify the conditions when state and trait measures do and do not correspond. 

Another way in which these measures of state emotional clarity vary across studies is in the length of the response scales and whether the scales were unipolar or bipolar. Momentary emotional clarity items frequently use 5-point scales (Bailen et al. 2019; Park and Naragon-Gainey 2019; Thompson and Boden 2019; Tuck et al. 2023) or 7-point scales (e.g., Eckland and Berenbaum 2021; Eckland and English 2023; Eisele et al. 2023; Springstein et al. 2023). Using a 5-point scale is consistent with three of the widely used trait emotional clarity scales (i.e., TMMS, TAS, and Difficulties in Emotion Regulation Scale [DERS; Gratz and Roemer 2004]), which use 5-point scales, whereas the Mood Awareness Scale (MAS; Swinkels and Giuliano 1995) uses a 6-point scale. Most of these studies examined momentary emotional clarity using a unipolar scale. That is, the left anchor of the scale indicated some variation of no emotional clarity (e.g., “not clearly at all”; Park and Naragon-Gainey 2019). In contrast, Eckland and Berenbaum (2021) presented participants with statements which were rated using a bipolar Likert scale (1 = disagree strongly, 7 = agree strongly). The TMSS and TAS also use bipolar Likert scales (i.e., rating agreement with a statement from strongly disagree to strongly agree), and the DERS uses a 5-point unipolar scale to assess how often an item applies to them (1 = almost never [0–10% of the time], 5 = almost always [91–100% of the time]). The literature on psychometrics suggests that there are reasons to prefer a 7-point scale over a 5-point scale (e.g., increased sensitivity; Finstad 2010). However, it is a complex issue, with some data suggesting 5- and 7-point measures produce nearly identical means, skewness, and kurtosis when rescaled to the same scale (Dawes 2008). A 5-point scale may be advantageous as it is more consistent with trait measures, possibly increasing the comparability of state and trait findings. Further, a 5-point scale has some practical advantages when assessing momentary emotional clarity in an experience sampling study (e.g., more likely to fit a mobile device screen). Thus, study design decisions should be weighed carefully to balance practical concerns, psychometric scale properties, and consistency with extant state and trait emotional clarity measures. 

The extent to which state emotional clarity depends on one’s trait levels may in part be reflected in the proportion of variance in state emotional clarity that is within-subjects (at the level of moments/situations) versus between-subjects (at the person level). Each of these studies examined the proportion of variance of the momentary emotional clarity was at the within- versus between-person levels using the intraclass correlation (ICC). Bailen et al. (2019) and Thompson and Boden (2019) both reported an ICC of .53 for their EMA item, meaning that 53% of the variance in momentary emotional clarity was at the between-person level and 47% of the variance was at the within-person level. Using the same item, both Springstein et al. (2023) and Eckland and English (2023) reported an ICC of .51 for their one-item emotional clarity measure and Tuck et al. (2023) reported an ICC of .40. Park and Naragon-Gainey (2019) reported an ICC of .34 for their 1-item emotional clarity measure. Finally, Eckland and Berenbaum (2021) found an ICC of .46 for their 2-item emotional clarity scale. Across these studies, about one-half to two-thirds of the variance in momentary emotional clarity was due to within-person variance, indicating that emotional clarity has a significant within-person component that fluctuates over time. 

A final concern regarding measuring state emotional clarity over the course of an experience sampling study is whether being asked to report on one’s emotions and one’s level of emotional clarity will systematically increase the emotional clarity over the course of the study. Eisele et al. (2023) report that, qualitatively, participants reported becoming more aware of their emotions during the experience sampling study. However, their quantitative analyses did not suggest that the emotional clarity increased during the experience sampling period. Like Eisele et al. (2023), Springstein et al. (2023) did not find any effects of time in the study on the levels of emotional clarity reported. Taken together, research has not found systematic time effects on emotional clarity, illustrating that participation in an experience sampling study is unlikely to increase one’s levels of emotional clarity.

**Figure 1:**
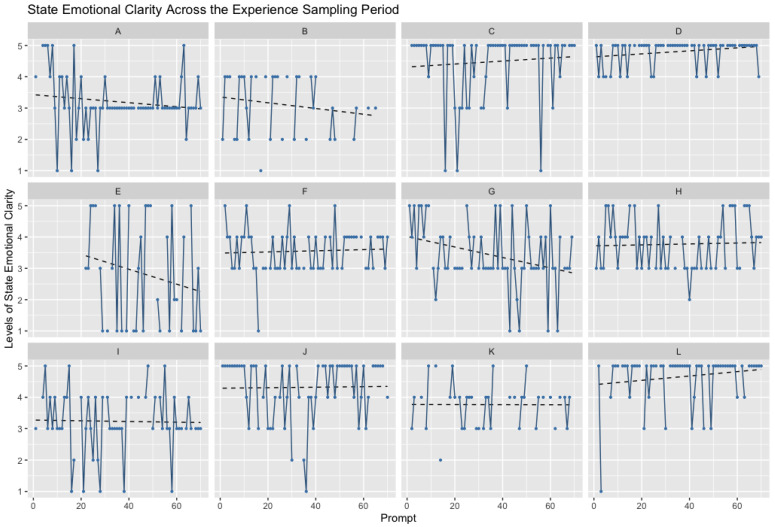
Levels of State Emotional Clarity across a Two-Week Experience Sampling period. Each panel (**A**–**L**) shows one participant’s levels of state emotional clarity reported during two weeks of experience sampling. Each dot represents an experience sampling prompt. Dotted lines represent lines of best fit for that participant’s levels of emotional clarity as a function of time. Across participants, consistent time effects do not emerge as a result of reporting emotional clarity during an experience sampling study.

To illustrate the lack of systematic time effects on momentary emotional clarity, we present data from 12 randomly selected participants from the authors’ most recent experience sampling study. Figure 1 shows the levels of state emotional clarity across 70 prompts of experience sampling (five prompts per day for 14 days). These data come from an unselected community sample of 18–65-year-olds responding to the item “During the last hour, my emotions were clear” using a 5-point bipolar Likert scale (1 = Strongly disagree, 5 = Strongly agree).

### 4.2. Indirect Measures

Indirect measures of state emotional clarity capture the performance or speed of information processing that is relevant to a target characteristic (Robinson and Neighbors 2006). These measures are especially useful when a target characteristic may be socially desirable (e.g., it may be socially desirable to be someone who is “emotionally intelligent”). 

Lischetzke et al. (2005) proposed and validated a measure of momentary emotional clarity that only relies on reports of current emotions. This measure of momentary emotional clarity is derived from the reaction time (RT) it takes for one to rate their emotions. Drawing on research using RTs as a measure of attitude strength (Bassili 1996) and work, demonstrating longer RTs for making judgments about ambiguous stimuli vs. unambiguous stimuli, they argue that RTs should be shorter for clearer, less ambiguous emotional experiences. Lischetzke et al. (2011) recommend statistically controlling for the baseline reading speed, and Thompson et al. (2015) controlled for the baseline RT to non-emotion items. This measure is related to trait measures of emotional clarity in some studies (Lischetzke et al. 2005, 2011), but not consistently in others (Thompson et al. 2015). In addition, it has the advantage of being parsed by valence (Thompson et al. 2015). 

Although RT has the advantage of being unobtrusive and less subject to desirability bias, this measure of state emotional clarity has some important limitations. First, clear data processing rules are needed when working with RTs (Lachaud and Renaud 2011); for example, rules for distinguishing between longer RTs due to low emotional clarity vs. inattention when completing the survey prompt. RTs gathered in daily life may also be noisier than those gathered in a controlled lab setting. Implicit measures, more broadly, tend to show lower test-retest reliability and greater temporal instability relative to the corresponding explicit measures (Gawronski et al. 2017). Thus, RT measures may reflect state emotional clarity to a lesser extent than they reflect trait emotional clarity.

### 4.3. Recommendations for State and Momentary Measures

When measuring state emotional clarity, several factors should be considered. Given the brevity of state and momentary measures, they are unable to capture the same amount of construct coverage that longer trait scales can. Consequently, measuring state emotional clarity using a combination of direct and indirect measures may be superior to using either in isolation. When using direct measures, single-item measures strongly limit how broadly and reliably a construct can be measured (Nunnally 1978), which ultimately limit its potential to predict important outcomes (Flake et al. 2017). Whenever possible, using more items to measure state emotional clarity can produce a more reliable, useful measure. Only one of the studies reviewed above included a measure of state clarity with more than one item (Eckland and Berenbaum 2021). One advantage of indirect measures is that they are relatively unobtrusive to collect. Thus, combining multiple measures (e.g., 1–2 direct questions, RTs to emotion items) may produce a stronger, multi-approach measure of state emotional clarity without increasing the burden in studies such as intensive longitudinal designs. For measures using multiple items, reporting between- and within-person reliability and a discussion of how the items were selected are essential steps for continuing to validate the construct of state emotional clarity across the literature. 

Though self-report items have typically been avoided in the assessment of emotional intelligence abilities, it may be necessary to include this method in bridging fluid and crystallized emotional intelligence. In avoiding a self-report methodology, the current measures of emotional intelligence ability (e.g., the MSCEIT, STEU) are unable to assess the experiential employment of emotional intelligence. Ortony et al. (2007) note that a critical implication of this gap in the assessment is that intelligent machines can use algorithms to score highly on measures of emotional intelligence without experiencing emotions at all. They urge researchers to expand the methods used to assess emotional intelligence to include self-report, informant-report, interviews, physiology, and behavior to capture a fuller understanding of emotional intelligence. 

## 5. Within-Person Variability in State Emotional Clarity

Within-person approaches to understanding psychological phenomena are becoming increasingly popular for explaining behavior (e.g., Dalal et al. 2020; Myin-Germeys et al. 2009). Empirical evidence suggests that phenomena previously considered to be “fixed”, like personality, are dynamic and fluctuate across time and situations. For example, for even the most (typically) extraverted person, some situations, like studying at a library, may produce trait-inconsistent behavior. However, it is not only personality traits that can be dynamic within persons—abilities, or access to one’s abilities, can also fluctuate in different settings. Cognitive abilities also appear to fluctuate within persons in daily life. Campbell et al. (2020) found that performance in ambulatory neurocognitive tests varied in daily life as a function of activities that the participants reported engaging in. Their results indicate that it may be easier to engage one’s cognitive abilities in a cognitively demanding task when one is already involved in mentally engaging activities (versus having to move from a state of disengagement to engagement). In other words, mentally engaging situations may facilitate access to one’s cognitive abilities. Within organizational psychology, Fit theory (van Vianen 2018) has also been used to explain how certain (e.g., work) environments facilitate the use of one’s abilities (i.e., person-environment fit), whereas others do not. Thus, it is reasonable to then expect that access to one’s emotional intelligence abilities may also be limited or enhanced across situations. 

As described above, the current body of studies examining state emotional clarity estimate that about one-half to two-thirds of the variance in emotional clarity is at the within-person level. This indicates that, on average, each person’s state or momentary emotional clarity varies across time and contexts. Fluctuations in momentary emotional clarity likely reflect the extent to which one’s abilities to be clear in the moment are either enhanced or hindered by factors such as the context in which emotions are unfolding. 

**Figure 2:**
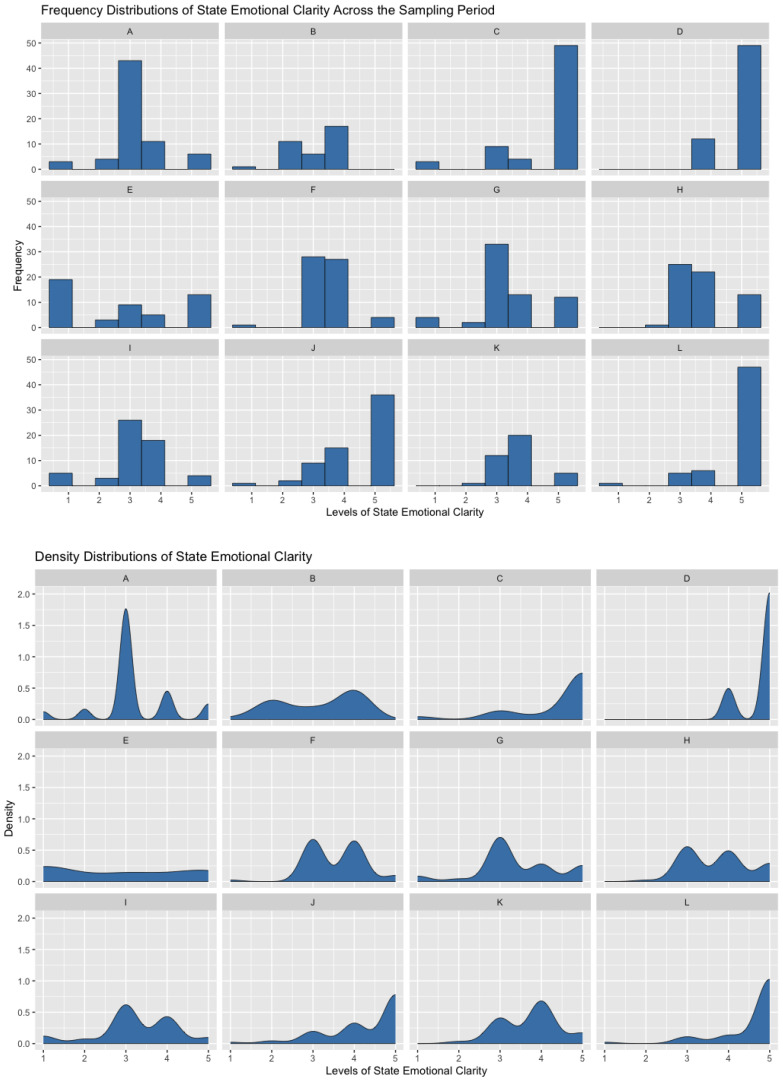
Frequency (**top**) and Density (**bottom**) Distributions of State Emotional Clarity across Two Weeks of Experience Sampling. Each panel (**A**–**L**) shows one participant’s frequency (**top**) and density (**bottom**) distributions of state emotional clarity reported during two weeks of experience sampling. Participants vary in the shape, center, and spread of reported levels of state emotional clarity across a two-week period. The same participants are shown in the top and bottom panel plots (e.g., Participant A’s frequency distribution is shown at the top and their density distribution is shown at the bottom).

The broader literature on personality traits and states provides some clues as to why people vary in emotional clarity and how to understand the relation between the trait and state levels. Whole trait theory (Fleeson and Jayawickreme 2015) argues two key points that may explain why emotional clarity fluctuates. First, traits are made up of the density distributions of states. People’s understanding of their emotions will vary moment-to-moment based on situational elicitors of emotional response. In some situations, people with typically high levels of emotional clarity will have great confusion about what they feel. Conversely, people with typically low levels of emotional clarity will, in some situations, clearly understand what they feel. Density distributions should also reflect individual differences in the trait level and states that individuals tend to experience. For those with less fluctuation in their state levels, their within-person standard deviations should be smaller. For those with higher trait levels of emotional clarity, their density distribution may have a negative skew (though higher trait levels could also be represented by a normal distribution with a higher mean). Figure 2 demonstrates the variability in the distributions of this ability in daily life with the frequency and density distributions of state emotional clarity across two weeks of experience sampling from the same randomly selected participants whose data are presented in Figure 1. 

A second assertion from whole trait theory that helps to characterize fluctuations in emotional clarity is that the stable mean of the density distribution reflects a descriptive trait level, while the spread of states reflects the influence of social-cognitive mechanisms (e.g., goals, beliefs, values) that rise in response to one’s situation/context. The process of clearly identifying one’s emotional state is one of signal detection (Klein and Robinson 2021). To perceive one’s emotional state, the emotional signal must be clearly identified amongst all the contextual noise around that signal. State (and momentary) emotional clarity in daily life then represents one’s ability to detect signals through the various sources of quotidian noise. Below, we describe several contextual factors that may enhance or mask emotional signals in daily life.

### 5.1. Contextual Factors Influencing Levels of State Emotional Clarity

#### 5.1.1. Affect Intensity

The extent to which one can clearly understands their emotions should be linked to how intense those emotions are. When examining RTs to emotion items, Thompson et al. (2015) found that both positive and negative emotional intensity were linearly associated with longer RTs (i.e., more intense affect was associated with less emotional clarity). Arndt et al. (2018) further tested this by including both linear and quadratic associations between emotional intensity and RTs to emotion items. They found that an inverse-U shaped curve best characterized this association. In other words, momentary levels of emotional clarity, as indexed by faster RTs to responding to emotion items, are higher when the emotional intensity is either lower or higher. Furthermore, Arndt et al. (2018) found that confidence in emotion ratings followed a U-shaped pattern when plotted against emotional intensity, such that people were more confident (i.e., clearer) at lower and higher levels of emotional intensity. Using face-valid emotional clarity items, Thompson and Boden (2019) replicated this pattern of association, such that emotional clarity was highest at lower and higher levels of emotional intensity. 

Emotional clarity should vary based on the intensity of the emotional signal that one is detecting (Klein and Robinson 2021). Clearer signals should be available at very low levels of affect intensity (i.e., detecting whether or not the signal is even present) and at very high levels of affect intensity (i.e., detecting ceiling levels of a signal). Thus, at more moderate levels of an emotion, emotional intensity may be a more ambiguous, less helpful, signal, and other factors may take precedence in determining the levels of state emotional clarity.

#### 5.1.2. Social Situations

Social interactions often elicit emotions, and emotions impact social interaction (Van Kleef 2016). Therefore, having a greater momentary understanding of one’s emotions should facilitate adaptive social behavior. Prospective longitudinal studies further suggest that deficits in emotional clarity are linked to poorer social functioning and maladaptive social behavior among adolescents (Rudolph et al. 2020). Two experience sampling studies (Thompson and Boden 2019; Tuck et al. 2023) indicate that momentary emotional clarity is higher during prompts where participants also reported having social interactions. Tuck et al. (2023) further elucidates this pattern by showing that the association between momentary emotional clarity and being in a social interaction is moderated such that momentary emotional clarity is even higher when interacting with close others. In social contexts, especially social contexts where one is highly motivated to maintain social harmony (e.g., with close others), people may be more motivated to understand their emotions as they are unfolding and make greater efforts to understand how they feel.

#### 5.1.3. Familiar Situations

Appraisal theories (e.g., Ortony et al. 1988) argue that emotions, in part, arise out of meaning that is made from situations. Among the many ways situations can be appraised is the extent to which they are experienced as familiar (versus unfamiliar). One’s mental representation of their current emotional state will be impacted by a variety of factors, including past feelings in similar situations (Barrett et al. 2007). Thus, in more familiar situations, emotions may become clearer because there is greater reliance on concepts such as how one typically feels in those situations. There is emerging direct empirical support for emotional clarity being higher in familiar situations. Two studies found that momentary emotional clarity is higher in more familiar situations in daily life (Springstein et al. 2023; Eckland and English 2023). 

#### 5.1.4. Sources of Emotions

State emotional clarity concerning the type of emotion one feels may in part depend on how clear the source of that emotion is. Boden and Berenbaum (2011) distinguished two types of emotional clarity: clarity of type (i.e., understanding the types of emotions one feels) and clarity of source (i.e., understanding the causes of one’s emotions). The vast majority of the emotional clarity literature focuses on emotional clarity of type. However, we argue that understanding the sources of emotions will help with identifying the types of emotions one feels. A wealth of studies indicate that being aware of the sources of information impacts how that information is processed (Keltner et al. 1993). For example, Schwarz and Clore (1983) demonstrate that unpleasant emotional information can impact judgments of life satisfaction, but this effect is mitigated by bringing the source of negative information into awareness. Appraisals of emotional sources differentially activate needs, goals, and concerns that impact the types of emotions people feel (Frijda 1986; Ortony et al. 1988; Siemer et al. 2007). For example, an argument with one’s spouse may elicit a variety of emotions, like anger or anxiety, but the specific type may depend on the concerns made salient by the argument. In the case of an argument with one’s spouse, identifying that one’s emotions are coming from feeling disrespected may help one identify that they are angry. On the other hand, identifying that the source of one’s feelings are thoughts such as “my spouse might leave me” may help one identify that they are fearful or anxious. Depending on the source and type of emotion, one may have different behavioral responses (e.g., taking a moment to cool off). Sources that are more ambiguous or difficult to interpret may lead to subsequent confusion about what one feels in the moment and what can be done about those feelings. 

#### 5.1.5. Interoceptive Cues

Interoception refers to the processing and representation of bodily signals (Quigley et al. 2021). Emotions involve physiological components, such as changes in heart rate, temperature, sweating, and muscle contractions/tension (Kreibig 2010). Individuals differ in their levels of interoceptive awareness and the accuracy with which they decipher interoceptive cues (Ludwick-Rosenthal and Neufeld 1985; Murphy et al. 2019). For those with greater interoceptive awareness, momentary emotional clarity may depend on appraisals of physiological changes. For example, a highly interoceptive person may detect increases in their heart rate and more clearly identify momentary levels of anxiety or excitement. However, physiological changes may signal a variety of emotions (e.g., increased heart rate could signal anxiety or excitement or both); thus, other contextual factors might be needed for clearly identifying what one feels in the moment. 

The usefulness of interoceptive cues may also depend on age. The physiological hypothesis of emotional aging (MacCormack et al. 2022) argues that, as part of the aging process, there is greater afferent noise from body signals to the brain. Therefore, the brain’s representations of emotions rely more on external cues and experience rather than interoceptive cues. As people get older, state and momentary levels of emotional clarity may depend less on interoceptive body cues.

#### 5.1.6. Significant Events

Functional theories of emotions (Keltner and Gross 1999) propose that emotions help to coordinate attention and action in response to events that are salient to one’s goals, needs, and values. Goal attainment (Emmons 1986), need fulfillment (Tay and Diener 2011), and value-congruent action (Luoma et al. 2007) have been linked to enhanced subjective well-being. Therefore, being able to understand one’s emotions in the moment should be helpful for facilitating action that promotes well-being. In line with this reasoning, Thompson and Boden (2019) found that following significant positive events, participants in an experience sampling study reported higher levels of momentary emotional clarity. In the context of significant events, people may have greater motivation to make sense out of their emotions in the moment to facilitate meaningful action.

### 5.2. Why Does Context Matter?

Understanding the variability and fluctuations in one’s ability to clearly understand their emotions has important implications. Over the last two decades, many efforts have been made to increase socioemotional skills and emotional intelligence abilities (e.g., including formal education in school settings; Durlak et al. 2011). In addition to interventions for socio-emotional skills, psychotherapy interventions such as cognitive behavioral therapy have demonstrated effectiveness for increasing the ability to clearly identify one’s feelings (Baker et al. 2012; Berking et al. 2013). Further identification of contexts that support, or inhibit, the ability to clearly identify emotions in the moment could enhance socio-emotional skills and psychotherapy interventions and provide greater specificity about circumstances when more effort may be needed to become clear.

## 6. Future Directions in State Emotional Clarity Research

Thus far, the research examining state emotional clarity has mostly focused on how momentary emotional clarity is related to the momentary experience of emotion. That is, the existing research has examined its associations with momentary negative and positive affect (e.g., Arndt et al. 2018; Lischetzke et al. 2011). Though this is an important area of work, state emotional clarity has relevance to other psychological processes that unfold in everyday life. Below, we discuss how state emotional clarity could be incorporated into the study of the other psychological processes that draw on one’s emotional intelligence abilities.

### 6.1. Emotion Regulation 

Prominent models of emotional intelligence include the successful management or regulation of emotions as a critical skill for emotionally intelligent people to have (Joseph and Newman 2010; Mayer et al. 2002). Despite a consistent designation of emotional clarity as part of the emotion regulation process, empirical work has yet to elucidate the specific ways in which emotional clarity enhances emotion regulation. Examining state emotional clarity, especially in daily life, may explicate how the ability to clearly perceive one’s emotions facilitates emotion regulation. Cybernetic models such as Larsen’s (2000) model of mood regulation suggest that a clear perception of emotions is needed for determining the need to regulate. The extended process model of emotion regulation (Gross 2015), another cybernetic model, also suggests that emotional clarity may be useful at several points in the emotion regulatory process, including in the identification of the need to regulate and in the selection of strategies to address one’s regulatory needs. Furthermore, Gratz and Roemer (2004) include deficits in emotional clarity as a contributor to difficulties in regulating emotions. Though these models do not explicitly refer to state or momentary emotional clarity, they imply that as the emotion regulatory process unfolds, a momentary understanding of emotions is critical for supporting successful emotion regulation.

In line with these theories, emerging evidence suggests that state emotional clarity has links with successful emotion regulation and coping in daily life. Park and Naragon-Gainey (2019) found that diminished momentary emotional clarity was associated with greater subsequent internalizing symptoms via less successful emotion regulation in an experience sampling study of people seeking treatment for internalizing disorders. Eckland and Berenbaum (2021) found that on days when participants reported greater emotional clarity than was typical for them, they also reported increased active coping with daily problems. Drawing upon these models of emotion regulation, we propose that future studies of emotional experience in daily life should continue to examine how momentary emotional clarity fits into the emotion regulation process. 

Both of the models by Larsen (2000) and Gross (2015) imply that momentary emotional clarity may help with determining needs to regulate or the decision of whether to regulate one’s emotions. Empirical work is needed to test whether momentary emotional clarity is associated with (a) one’s appraisals of their need to regulate emotions in the moment and (b) decisions not to regulate emotions. Gross (2015) further describes the possibility that momentary emotional clarity may facilitate strategy selection. Thus, empirical work is needed to test whether momentary emotional clarity helps with selecting strategies that fit one’s context/situation, regulation needs (i.e., the strategy is effective for the specific emotion being regulated), or adjusting the use of strategies that are not working. Finally, further replication is needed of state/momentary emotional clarity’s link to emotion regulation success, including testing the boundaries of this effect. Is emotional clarity always needed in the moment to successfully regulate emotions? Under what conditions does momentary emotional clarity contribute to emotion regulation success or not?

### 6.2. Decision Making 

Under the four-branch model (Mayer et al. 2002), using emotions to facilitate thinking processes (e.g., decision making) is considered a marker of emotionally intelligent people. Several theoretical models and empirical accounts demonstrate that momentary emotional clarity can help with decisions of whether to regulate one’s emotions or not. Larsen’s (2000) control-process model of mood regulation suggests that emotional clarity is needed for recognizing discrepancies between one’s current and baseline mood. Discrepancies are compared with one’s current goals or concerns so that one can decide whether to engage in mood regulating behavior. In the extended process model (Gross 2015), emotional clarity is thought to be important for both determining a need to regulate (similar to Larsen’s model) as well as in deciding which strategy to engage in. Emotion is thought to serve as a barometer of one’s goal progress (Carver and Scheier 1990); therefore, a clear understanding of one’s emotions should facilitate making decisions to continue or re-orient goal pursuit. Having higher trait and momentary emotional clarity should have implications for the availability of cognitive resources. Higher trait emotional clarity would allow one to use fewer resources in the moment to determine one’s feelings, while higher momentary emotional clarity allows for more cognitive resources to be devoted to responding to the demands of one’s situation. 

Emotions that are integral (i.e., related to a decision-making situation) and incidental (i.e., present during a decision-making situation, but unrelated to the situation itself) have both been shown to impact the decision-making process (Lerner et al. 2015). In situations where the emotion is integral to the decision, momentary emotional clarity may facilitate making decisions with greater speed or certainty because situation-relevant information from one’s emotions is more easily available. Lab-based studies have demonstrated that people with a greater understanding of their emotions show less bias in their decision making from incidental emotions (Yip and Côté 2013). Future research should test the generalizability of these findings to everyday life. For example, does state emotional clarity in daily life have the same buffering effects against bias from incidental emotions in everyday decisions? For what types of decisions (e.g., the personal relevance, the importance of the decision) is state/momentary emotional clarity helpful? In what types of situations (e.g., ambiguous, social, low-risk) is state/momentary emotional clarity especially helpful?

### 6.3. Goals 

Behavior is directed by goals (internal representations of desired states; Austin and Vancouver 1996). Dweck (2017) proposes that our day-to-day goals stem from core psychological needs and the fulfillment of those goals promotes our psychological well-being. Carver and Scheier (1990) applied a control-process model to goal pursuit and suggested that emotions arise to help in judging discrepancies between one’s desired state (i.e., their goal) and their current state. When our goals/needs are met, pleasant emotion is elicited. When our goals/needs are not being met, unpleasant emotion is elicited. Emotions can serve as a barometer of whether our goals are being met or whether we are moving closer or further away from meeting our goals (Carver and Scheier 1990; Larsen 2000). Momentary levels of clarity should therefore be helpful in pursuing and attaining goals.

The affect-as-information approach (Clore et al. 2001; Gohm 2003; Gohm and Clore 2002; Storbeck and Clore 2008) suggests that our affective (e.g., emotional, mood, liking/disliking) reactions provide salient information about a range of important considerations, such as our environment, situation, and/or goal progress. Thus, what someone understands about their emotions may provide a range of information about their goals. For example, could low state emotional clarity (i.e., one understands that they feel “bad” but may not be able to identify their emotions more specifically) lead one to conclude that they are not making progress in their goal? Alternatively, does higher momentary emotional clarity of negative affect (e.g., being able to clearly identify frustration in the moment) lead one to conclude that they need to change their approach to the goal?

## 7. Conclusions

In contrast to the substantial body of work examining trait emotional clarity, the literature surrounding state emotional clarity is still in its nascency. While the research on trait emotional clarity has established its importance for well-being, psychopathology, and many psychological processes, we view research examining state and momentary emotional clarity as a crucial next step in integrating emotional intelligence abilities into the study of psychological processes in daily life. Research further testing contexts which support (or inhibit) this ability and the downstream effects that being clear in the moment have will be important for understanding how to best cultivate the ability to be emotionally clear.

## Data Availability

No new data were created or analyzed in this study. Data sharing is not applicable to this article.
